# Protein lysine acetylation does not contribute to the high rates of fatty acid oxidation seen in the post-ischemic heart

**DOI:** 10.1038/s41598-024-51571-0

**Published:** 2024-01-12

**Authors:** Ezra B. Ketema, Muhammad Ahsan, Liyan Zhang, Qutuba G. Karwi, Gary D. Lopaschuk

**Affiliations:** Cardiovascular Research Centre, Department of Pediatrics, University of Alberta, 423 Heritage Medical Research Centre, Edmonton, AB T6G 2S2 Canada

**Keywords:** Cardiology, Cardiovascular diseases

## Abstract

High rates of cardiac fatty acid oxidation during reperfusion of ischemic hearts contribute to contractile dysfunction. This study aimed to investigate whether lysine acetylation affects fatty acid oxidation rates and recovery in post-ischemic hearts. Isolated working hearts from Sprague Dawley rats were perfused with 1.2 mM palmitate and 5 mM glucose and subjected to 30 min of ischemia and 40 min of reperfusion. Cardiac function, fatty acid oxidation, glucose oxidation, and glycolysis rates were compared between pre- and post-ischemic hearts. The acetylation status of enzymes involved in cardiac energy metabolism was assessed in both groups. Reperfusion after ischemia resulted in only a 41% recovery of cardiac work. Fatty acid oxidation and glycolysis rates increased while glucose oxidation rates decreased. The contribution of fatty acid oxidation to ATP production and TCA cycle activity increased from 90 to 93% and from 94.9 to 98.3%, respectively, in post-ischemic hearts. However, the overall acetylation status and acetylation levels of metabolic enzymes did not change in response to ischemia and reperfusion. These findings suggest that acetylation may not contribute to the high rates of fatty acid oxidation and reduced glucose oxidation observed in post-ischemic hearts perfused with high levels of palmitate substrate.

## Introduction

Ischemic heart disease is a leading cause of cardiovascular deaths globally, affecting over 126 million people and resulting in more than 9 million deaths annually^1^. Myocardial ischemia occurs when blood flow to the heart is obstructed due to either a partial or complete blockage of coronary arteries or reduced coronary blood flow^2,3^. Although successful therapeutic interventions have been developed to minimize ischemic injuries and cell death (infarction) through timely restoration of blood flow (reperfusion)^4,5^, reperfusion itself can also induce additional irreversible injury and death in heart tissue that survived the ischemic insult^4,6^. Clinical studies have also demonstrated a high risk of heart failure development and mortality in ischemic survivors despite reperfusion therapies^7,8^, suggesting limitations in the available treatments.

One contributing factor to injury and contractile dysfunction during reperfusion of the ischemic heart is the high rates of fatty acid oxidation observed during reperfusion^9–12^. This phenomenon can be attributed to at least two main factors. Firstly, circulating levels of fatty acids are elevated after cardiac bypass surgery or myocardial infarction^13–17^ due to adrenergic stimulation of adipose tissue lipolysis^18,19^. This elevated fatty acid supply increases cardiac fatty acid oxidation during reperfusion. Secondly, cardiac mitochondrial fatty acid uptake is increased during reperfusion after ischemia due to decreased malonyl-CoA levels^20,21^. Malonyl CoA is an important inhibitor of carnitine palmitoyl transferase-1 (CPT-1), the key enzyme for mitochondrial fatty acid transport in the heart^22^. Cardiac malonyl CoA levels decrease during ischemia and reperfusion due to an ischemic-induced activation of AMP-activated protein kinase^20^, leading to uncontrolled mitochondrial fatty acid uptake and β-oxidation^21,23^. Both circumstances lead to increased delivery and utilization of fatty acids in the myocardium during reperfusion after ischemia.

High fatty acid oxidation rates during reperfusion of ischemic hearts contribute to contractile dysfunction^10,11,24,25^ and have also been shown to contribute to infarct size and mortality in patients^26,27^. The negative effects of excessive fatty acid usage as the sole fuel source are associated with an inefficient oxygen utilization and inhibition of myocardial glucose oxidation^10,11,28^. The suppression of glucose oxidation occurs in the presence of accelerated anaerobic glycolysis, leading to the uncoupling of glycolysis from glucose oxidation^29^. This uncoupling results in lactate accumulation and excess proton (H^+^) production, causing disturbances in intracellular Na^+^ and Ca^2+^ homeostasis, further worsening contractile dysfunction and post-ischemic injury^30,31^.

Despite being known for decades, effective therapeutic interventions to reverse the imbalanced fuel use in post-ischemic hearts are still limited in clinical practice. Changes in the lysine acetylation status of fatty acid metabolic enzymes is one of the regulatory mechanisms controlling flux through mitochondrial metabolic pathways^32–34^. Studies by our group and others have shown that acetylation of fatty acid oxidation enzymes results in an increased myocardial fatty acid oxidation in animal models of chronic high-fat diet and diabetes^33,35,36^. In contrast, acetylation decreases glucose oxidation by inhibiting the pyruvate dehydrogenase enzyme^37,38^. These data suggest that increased protein acetylation could contribute to the metabolic inflexibility seen in heart failure through the differential regulation of metabolic enzyme activities. In contrast, a recent study by Davidson et al. dismissed the role of mitochondrial protein hyperacetylation in transverse aortic constriction (TAC) induced heart failure^39^. Using a genetic model of markedly elevated cardiac mitochondrial protein hyperacetylation, the authors observed no significant impact of protein lysine acetylation changes on mitochondrial function and heart failure progression after TAC^39^. However, the acetylation status and activities of major mitochondrial metabolic enzymes were not specifically assessed in this study.

It is largely unknown what impacts myocardial I/R could have on mitochondrial protein lysine acetylation, and whether acetylation changes contribute to the cardiac energy metabolic shifts observed during myocardial ischemia and reperfusion (I/R) is not known. Myocardial ischemia has the potential to induce metabolic changes that could also enhance protein hyperacetylation. Firstly, a decrease in NAD^+^ levels as well as the downregulation of its salvaging enzyme, nicotinamide phosphoribosyl transferase (NAMPT), have been reported in myocardial I/R^40,41^. As NAD^+^ is a co-substrate for sirtuin deacetylase enzymes, including SIRT1 and SIRT3, decreased NAD^+^ levels during ischemia may reduce sirtuin activities, thereby causing hyperacetylation^42^. In addition, SIRT3 expression could also be downregulated following ischemia and reperfusion^43,44^. Secondly, increased myocardial fatty acid oxidation during reperfusion could also lead to the accumulation of mitochondrial acetyl-CoA, a substrate for protein lysine acetylation. However, although some studies have indicated the general susceptibility of hearts from sirtuin (deacetylase) deficient mice to ischemic injuries^45,46^, the role of acetylation dysregulation of fatty acid oxidative enzymes in fatty acid oxidation in post-ischemic hearts has not been studied. We, therefore, determined whether cardiac lysine acetylation changes during ischemia and reperfusion, and whether this has a role in increasing fatty acid oxidation in the post-ischemic heart.

## Methods

### Ethical approval and animal care

Study protocols for using Sprague–Dawley rats were reviewed and approved by the Animal Care and Use Committee at the University of Alberta. All animal experiments were conducted in compliance with the guidelines set by the Canadian Council of Animal Care. The study methods and results are reported in accordance with the ARRIVE guidelines^47^. Rats were housed in a temperature-controlled animal facility with a 12-h light/dark cycle. Additionally, the rats were provided ad libitum access to water and a regular chow diet. To determine the appropriate sample size and statistical power, we used G*Power software (v3.1.9.4, Heinrich-Heine-Universität Düsseldorf, Düsseldorf, Germany)^48^. The following factors were taken into consideration for the calculation. Five percent significance level (alpha or type-I error) (two-tailed), 80% power, effect size, as well as statistical tests used in the study (paired *t*-test or one-way ANOVA). For effect size estimation, we used the mean and standard deviation differences obtained from past studies under comparable conditions^49,50^. Computing this information into the G* power software yielded a total required sample size of 51 rats. These rats were allocated among three different groups: the aerobic control (n = 10), the end of ischemia groups (n = 7) and the ischemia and reperfusion group (n = 34).

### Isolated working rat heart perfusions

Isolated working rat heart perfusions were performed as previously described^10,51^. Briefly, after an intraperitoneal injection of sodium pentobarbital (60 mg/kg), hearts from male Sprague–Dawley rats (275 to 300 g) (Charles River, Kingston, Canada) were rapidly excised and immersed in ice-cold Krebs–Henseleit bicarbonate (KHB) solution. The aorta was isolated and cannulated, and the hearts were immediately perfused in the Langendorff mode (at 60 mmHg) with KHB solution containing (in mmol/l): NaCl 118, KCl 4.7, MgSO_4_ 7H_2_O 1.2, KH_2_PO_4_ 1.21, CaCl_2_H_2_O 2.4, glucose 5, and NaHCO_3_ 25, (pH 7.4). The KHB solution was gassed with 95% O_2_ and 5% CO_2_, and the temperature was maintained at 37 °C. During this time, excess tissues covering the heart (e.g. pericardium, lung, trachea, etc.) were removed, and the left atrium opening was cannulated via the pulmonary vein. The heart was then switched to the working mode by clamping the aortic inflow line from the Langendorff reservoir and opening the preload and after-load lines. Subsequently, the heart was placed in a glass chamber with the lower part prefilled with a 100 ml recirculating modified KHB solution containing 1.2 mM palmitate bound to 3% bovine serum albumin (BSA) (Equitech-Bio Inc, Kerrville, TX), 5 mM glucose, and 100 μU/ml insulin (Eli Lilly Inc, ON, Canada). The KHB solution also included appropriately radio-labeled substrates, [U-^14^C]-glucose, [5-^3^H]-glucose or [9,10-^3^H]-palmitate (Perkin Elmer, Boston, MA) for the measurement of glucose oxidation, glycolysis or fatty acid oxidation, respectively. The aortic and coronary outflows were recirculated through a closed air-tight system to allow the collection of ^14^CO_2_ from glucose (see below for details). Left atrial preload and aortic after-load were set at column heights equivalent to 11.5 mmHg and 80 mmHg, respectively.

### Assessment of ex vivo cardiac function

Hearts were perfused in the working mode and unpaced^51^. Heart rate, peak systolic pressure, and left ventricular developed pressure were measured using a Gould P21 pressure transducer (Harvard Apparatus, Holliston, MA) connected to the aortic outflow line. Data were acquired using an MP100 system from AcqKnowledge (BIOPAC Systems Inc., California). Cardiac output and aortic flow rates (ml/min) were obtained by measuring the perfusate flow rate into the left atria and from the after-load lines using Transonic T206 ultrasonic flow probes (Transonic Systems Inc., New York), respectively. Coronary flow (ml/min) was calculated as the difference between cardiac output and aortic flow. All data were collected at 10 min intervals. Cardiac work was determined as a function of cardiac output and peak systolic pressure and was calculated as (joules^.^min^–1.^g dry wt^–1^) = (peak systolic pressure (PSP)–11.5) × 133.322 x cardiac output (CO) × 0.000001 × 60/60. A conversion factor of 1.333 × 10^–4^ was used to convert cardiac power values from mmHg per ml to joules.

### Ischemia–reperfusion protocol

Hearts were initially perfused aerobically for 30 min, followed by 30 min of global no-flow ischemia and then 40 min of aerobic reperfusion as outlined previously^52^. Global no-flow ischemia was induced by clamping off both the left atrial and aortic flow lines for 30 min. Following 30 min of no-flow ischemia, left atrial and aortic flows were restored, and the recovery of mechanical function was monitored for a further 40 min period. Functional data and metabolic samples were collected every 10 min before and after ischemia. At the end of each perfusion, hearts were snap-frozen with liquid nitrogen, weighed, and then stored at − 80 °C. After grinding the frozen heart tissue at freezing temperature, a portion of the tissue (up to 30 mg) was weighed and dried in the oven for 24 h to remove its water content. The ratio of dry to wet weight was also calculated.

### Measurement of energy metabolic rates

For the determination of glycolysis, glucose oxidation and palmitate oxidation rates, the hearts were perfused with radioactive substrates: [ 5-^3^H]-glucose, [U-^14^C]-glucose or [9,10-^3^H]-palmitate, respectively. The flux rate for each substrate was determined by quantitatively collecting either myocardial ^3^H_2_O or ^14^CO_2_ produced during the perfusion period at 10 min intervals (see details below), as previously described^30^. The amount of radioactive ^3^H_2_O and ^14^CO_2_ was then quantified using a liquid scintillation counter (Beckman Coulter, LS6500). Buffer samples were collected through an injection port inserted between the buffer reservoir and the oxygenator. All samples were collected in duplicate at each time point. Metabolic rates were normalized to the dry mass of the heart to correct for variations in heart size. To minimize variation due to contractile performance, metabolic rates were also normalized to cardiac work. Glucose or fatty acid oxidation rates were expressed as nanomoles (nmol) of substrate oxidized per minute per gram dry heart weight. Glycolysis was expressed as nmol of glucose passing through glycolysis per minute per gram dry heart weight.

For the determination of glucose oxidation rates, the amount of ^14^CO_2_ released from U-^14^C glucose oxidation both into the perfusate and hyamine solution (in gaseous form) was quantified. The gaseous ^14^CO_2_ released into the perfusate was directed to the air outlet through specialized tubing until trapped in 30 ml of 1 M hyamine hydroxide solution (PerkinElmer, Waltham, MA). Subsequently, 150 µl of the hyamine sample was taken and mixed with the vial containing 4 ml of scintillation fluid. In addition, the ^14^CO_2_ dissolved in the form of H^14^CO^-^_3_ in the perfusate was extracted by injecting 1 ml of the perfusate sample into a 25-mL stoppered metabolic vial with a center hole on the top and containing 1 ml of 9 N H_2_SO_4_. The ^14^CO_2_ released after the reaction was trapped on hyamine hydroxide-soaked filter paper (300 µl) in a scintillation vial, which was flipped upside down and fitted into the rubber stopper to seal the center hole. Following overnight incubation, the scintillation vial with the filter paper was then taken and filled with 4 ml of scintillation fluid for quantification of radioactivity in the liquid scintillation counter.

Glycolysis and palmitate oxidation rates were measured by the rate of appearance of ^3^H_2_O in the perfusate from either ^5^H-glucose or [9,10-^3^H] palmitate, respectively, using a vapor transfer method^53^. Briefly, 500 μl of water was added into a 7 ml scintillation vial, followed by the insertion of a capless 1.5 ml microcentrifuge tube. Then, a 200 μl perfusate sample was added to the microcentrifuge tube placed inside the scintillation vial and incubated at 50 °C for 24 h to allow vaporization. It was further cooled at 4 °C for another 24 h before wiping all the water droplets on the outer wall of each capless tube into scintillation vials and discarding the capless tube with its content. The ^3^H_2_O transferred to the scintillation vial was quantified after the addition of 4 ml Ecolite scintillation fluid (MP Biomedicals, Solon, OH) in a liquid scintillation counter. The individual measurements were normalized for transfer efficiency, which was determined as the ratio between vaporized ^3^H_2_O together with the samples and the specific activity of unmetabolized (unprocessed) ^3^H_2_O.

### ATP production rates and tricarboxylic acid cycle (TCA) activity

To determine the contribution of glucose and palmitate oxidation to the overall tricarboxylic acid (TCA) cycle activity, we calculated the amount of acetyl-CoA derived from glucose and palmitate oxidation rates. A value of 2 acetyl-CoAs per molecule of glucose oxidized and 8 acetyl-CoAs per molecule of palmitate oxidized was used. Total ATP production rates from glucose oxidation, palmitate oxidation and glycolysis were determined by assuming 29 mole ATP produced per mole of glucose oxidized, 105 mol ATP produced per mole of palmitate oxidized, and 2 mol of ATP produced per mole of glucose passing through glycolysis.

### Tissue extraction and western blotting

Approximately 20–30 mg of frozen heart tissue was weighed and homogenized in ice-cold lysis buffer containing the following concentrations (mM): 50 Tris–HCl (pH 7.5), 5 ethylenediaminetetraacetic acid (EDTA), 0.5 ethylene glycol-bis(β-aminoethyl ether)-N,N,N′,N′-tetra acetic acid (EGTA), 150 NaCl, 5 nicotinamide, and 10 sodium butyrate. Additionally, the buffer contained 1% Triton X-100, 0.5% NP-40, 0.1% sodium dodecyl sulfate (SDS), 1% phosphatase inhibitor cocktail 1, 1% phosphatase inhibitor cocktail 2, 0.2% protease inhibitor and 0.02% trichostatin A (TSA). The samples were homogenized using a tissue homogenizer (OMNI International, Kennesaw GA) for 30–40 s in two rounds, followed by centrifugation at 10,000×*g* for 10 min. The resulting supernatant was collected, and the protein concentration was determined using Bio-Rad Protein Assay dye (Bradford reagent) (Bio-Rad #5000006) in the BioTek Synergy Mx Microplate Reader. Subsequently, protein aliquots were mixed with Laemmli buffer containing 50% glycerol, 2% SDS, 0.0025% bromophenol blue, 50 mM Tris–HCl (pH 6.8) with 16% (v/v) β-mercaptoethanol (BME) and heated at 80 °C for 7 min prior to separation by SDS–polyacrylamide gel electrophoresis (SDS-PAGE).

Protein lysate samples (equivalent to 30 µg of protein) were loaded and separated by 10% SDS-PAGE. The separated proteins were then transferred onto nitrocellulose membranes overnight at a cold temperature. Subsequently, the membranes were blocked with 5% skim milk for 1 h at room temperature followed by two 10 min washes with 1 × Tris-buffered saline containing 0.1% Tween-20 detergent (TBST). The membranes were then probed with one of the following primary antibodies overnight: pan acetyl-lysine (9441 s, Cell Signaling Technology (CST)), pan lysine succinyl (PTM-401, PTM Biolabs), SIRT3 (5490 s, Cell Signaling Technology (CST)), PDH (3205 s, Cell Signaling Technology (CST)), LCAD (ab128566, Abcam), β-HAD (ab37673, Abcam), glycerol-3-phosphate dehydrogenase (ab80535, Abcam), PGAM2 (PA5-98051, ThermoFisher Scientific), and α-tubulin (T6074, Millipore Sigma). Afterwards, the membranes underwent two 10 min washes with 1 × TBST and were then incubated with the appropriate secondary antibodies (1:5000) for 1 h at room temperature. The protein bands were visualized using an Amersham enhanced chemiluminescence kit (Cell Signaling Technology (CST), Danvers, MA) and were imaged by autoradiography. Finally, the intensity of the protein bands were quantified through densitometry analysis using ImageJ software (NIH, US) and normalized to the appropriate loading control.

### Immunoprecipitation

To investigate the changes in the acetylation status of key proteins involved in fatty acid and glucose metabolism, frozen heart tissue was homogenized as described above. Then, approximately 300 μg of protein lysate was pre-cleared using 50 μl of protein A/G-agarose beads (sc-2003, Santa Cruz Biotechnology, Inc.) for 1–2 h with gentle rotation. Next, the mixture was centrifuged at 3000×*g* for 10 min and the resulting supernatant was transferred into a new tube. To this, 2 μl of acetyl-lysine antibody (Cell Signaling Technology (CST), 9441) was added and incubated overnight at 4 °C with gentle agitation. The next day, 50 μl of A/G-agarose beads was added to each sample to pull down protein-antibody complexes. This mixture was then incubated for an additional 6–8 h under the same conditions. The samples were then centrifuged at 5000×*g* for 10 min to separate the bead-antibody-protein complex from the supernatant, which was discarded afterwards. The pellet was washed 3 times with the same lysis buffer used for homogenization with centrifugation at 5000×*g* for 10 min in between. Finally, the bead-antibody-protein complex was resuspended with 1 × Laemmli buffer and heated at 95 °C for 5 min to release the acetylated proteins from the antibody-bead complexes. Then, the samples were centrifuged at high speed (15,000×*g* for 12 min), and the supernatant containing acetylated protein was collected in new tubes. Western blot was then carried out to detect various acetylated protein targets. The proteins were separated by 10% SDS-PAGE, as described above. Both a negative control, A/G PLUS-Agarose beads bound to an anti-acetyl-lysine antibody without a protein sample, and a positive control, protein lysate sample without A/G PLUS-Agarose beads, were used. All the bands were normalized to the IgG heavy chain.

### NAD^+^ and NADH quantification

The concentration of nicotinamide adenine dinucleotide (NAD^+^) and its reduced form NADH in left ventricle tissue lysate samples was determined using the Amplite™ Colorimetric NADH Assay Kit (AAT Bioquest, Sunnyvale, CA; cat #15273) following the guidelines provided by the manufacturer. Briefly, the Amplite™ NAD/NADH Ratio Assay Kit contains the NADH probe, a chromogenic sensor with maximum absorbance at 460 nm upon NADH reduction. The absorbance increases proportional to the concentration of NADH in the sample. The NADH probe detects NADH in an enzyme-free reaction, and the absorbance was read in a microplate reader at 460 nm. For NAD^+^ quantification, 25 µl of NAD Extraction Solution was added to the test samples, followed by Neutralization Solution. The mixture was then incubated for 15 min before the absorbance was read at 460 nm.

### Statistical analysis

A paired, two-tailed Student’s *t* test, and one-way or two-way analysis of variance (ANOVA) were used to determine the statistical significance differences between pre-and post-ischemic values. An unpaired Student’s *t* test was used to assess significant differences between experimental groups. The data are presented as mean ± standard deviation (SD) unless otherwise specified. All statistical analyses were performed using GraphPad Prism V9 software (San Diego, CA). Differences were considered statistically significant when P < 0.05.

## Results

### Recovery of cardiac function is significantly impaired during aerobic reperfusion of ischemic hearts

**Figure 1:**
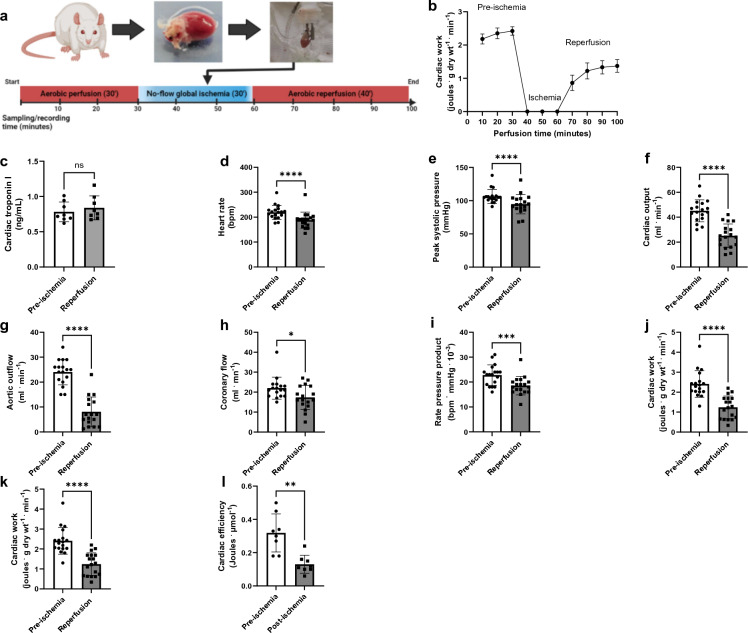
Incomplete recovery of mechanical function in isolated working rat heart after 30 min of global no-flow ischemia and 40 min of reperfusion. All hearts were stabilized and perfused aerobically for 30 min before ischemia. A total of 34 rat hearts were subjected to I/R protocol, from which 18 (~ 53%) of them recovered after ischemia. The hearts were perfused with 1.2 mM palmitate, 5 mM glucose, 100 μU/ml insulin, and radio-labeled substrates (**a**) Study protocol, (**b**) Recovery of cardiac work, (**c**) Cardiac troponin levels (cTn-I), (**d**) Heart rate, (**e**) Peak systolic pressure, (**f**) Developed pressure, (**g**) Cardiac output, (**h**) Aortic outflow, (**i**) Coronary flow, (**j**) Rate pressure product, (**k**) Cardiac work, and (**l**) Cardiac efficiency (n = 18: (**b**,**d**-**i**); n = 8: (**b**,**l**)). A Paired *t* test was used for pre-and post-ischemic comparisons (**c**–**k**), while a simple linear regression was used to demonstrate the changes in functional recovery over time (**b**). Data are expressed as mean ± SD. ****P < 0.0001,***P < 0.001, **P < 0.01 compared to pre-ischemic values.

To analyze the relationship between the recovery of mechanical function and the metabolic phenotype in post-ischemic hearts, we first characterized the severity of mechanical dysfunction following 30 min of no-flow global ischemia. The recovery rate after 30 min of no-flow ischemia was approximately 53%. Hearts that did not recover (unable to overcome the after-load pressure), (47% or 16/34), after the initial 10 min of reperfusion were not included in the final analysis. The 30 min global no-flow ischemia resulted in incomplete recovery for most of the parameters assessed (Fig. 1). Following reperfusion, cardiac work and efficiency declined by 41% and 49%, respectively, compared to pre-ischemic values. Despite a gradual improvement observed over the 40 min reperfusion period, both parameters failed to achieve full recovery (Fig. 1b). After 30 min of ischemia, the hearts recovered only 59% of cardiac output and 43% of aortic outflow rates. Similarly, the recovery rates for most other parameters remained below 80% of their pre-ischemic values (Fig. 1c-k).

### Fatty acid oxidation dominates cardiac energy metabolism in post-ischemic heart

**Figure 2:**
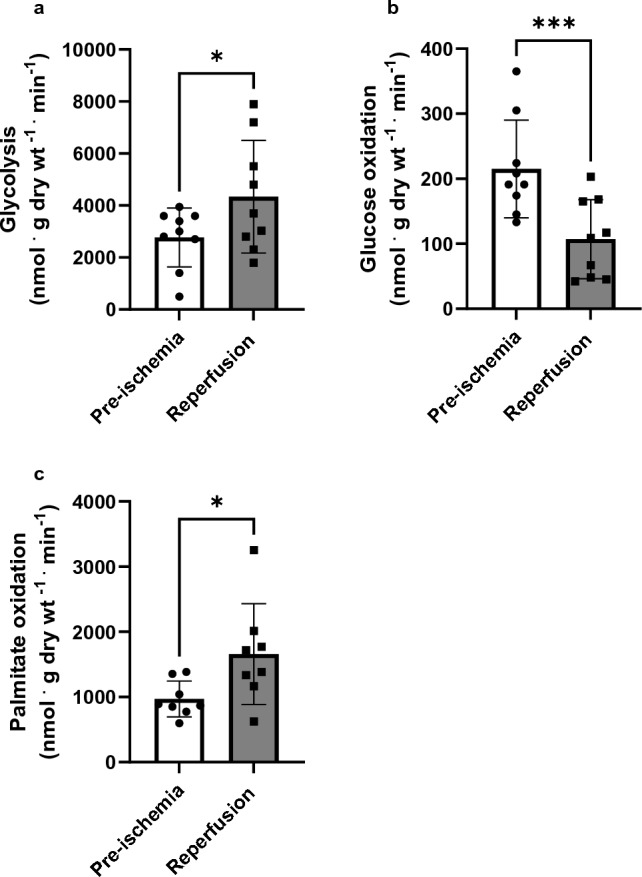
Impaired cardiac energy metabolism in post-ischemic rat hearts. The hearts were perfused with 1.2 mM palmitate pre-bound with % BSA, 5 mM glucose, and 100 μU/ml insulin (n = 12–13). ^3^H_2_0 production from [5-^3^H]-glucose and [9,10-^3^H] palmitate were used to determine glycolysis and palmitate (fatty acid) oxidation rates, while ^14^CO_2_ released from [U-^14^C]-glucose was used to determine glucose oxidation rates. Samples were collected at 10 min intervals before the ischemia and during the reperfusion. (**a**) Glycolysis (n = 9), (**b**) Glucose oxidation (n = 9), (**c**) Palmitate oxidation (n = 8). A Paired *t* test was used for pre and post-ischemic comparisons. Data are expressed as mean ± SD. ***P < 0.001, *P < 0.05 compared to pre-ischemic values.

Following 30 min of ischemia, reperfused hearts displayed a statistically significant rise in glycolysis rate compared to the pre-ischemic baseline values, showing a 1.5 fold higher increase (2772 vs 4336 nmol ^.^ g dry wt^-1^^.^ min^–1^, p < 0.0001). This increase was further amplified to 295% when normalized to cardiac work (1295 ± 563 vs 5127 ± 4175 nmol ^.^ joules^–1^) (Fig. 2a, Table 1). Conversely, the rates of glucose oxidation significantly decreased after reperfusion from 215.1 ± 75.16 to 107.1 ± 60.8 nmol ^.^ g dry wt^–1^^.^ min^–1^, p < 0.05, dropping by 51% from baseline values (Fig. 2b). This trend persisted after normalization to cardiac work (− 28%, p < 0.05) (Table 1). In contrast, there was a remarkable 71% surge in the rates of palmitate oxidation during the post-ischemic period compared to the baseline (970.4 ± 275.8 vs 1658.8 ± 773.2 nmol ^.^ g dry wt^-1^^.^ min^–1^, p < 0.05) (Fig. 2c). This increase was further accentuated upon normalization to cardiac work, exhibiting a substantial rise of 175% after reperfusion (Table 1, Supplement Fig. S1).

**Table 1:** Glycolysis, glucose oxidation, and palmitate oxidation rates normalized to cardiac work (nmol ^.^ joules^–1^) before and after ischemia in isolated working rat hearts.

Conditions	Glycolysis (n = 9)	Glucose oxidation (n = 9)	Palmitate oxidation (n = 8)
Baseline	1295.56 ± 563.41	83.47 ± 35.26	498.13 ± 173.37
Reperfusion	5127.78 ± 4175.42	62.43 ± 36.38	1608.88 ± 1243.93

**Figure 3:**
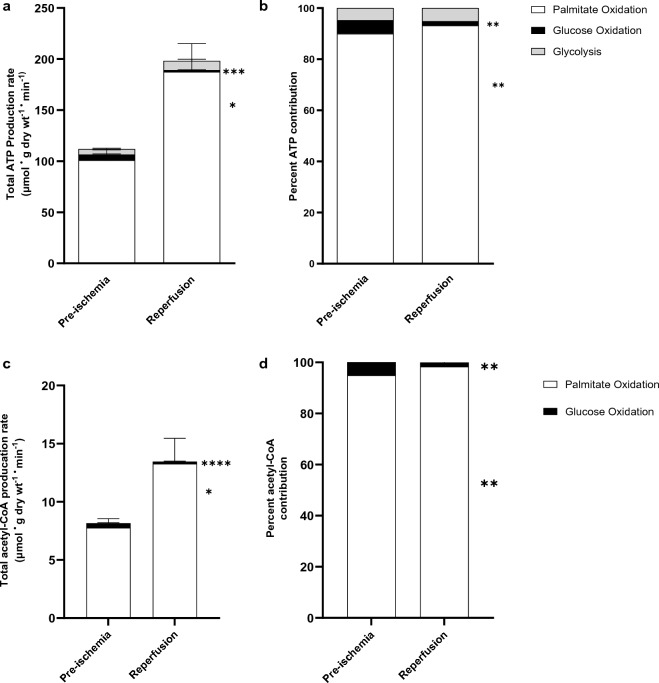
Reduced contribution of glucose oxidation to the total ATP production and TCA activity in post-ischemic rat heart. Cardiac adenosine triphosphate (ATP) production rate and TCA cycle activity. (**a**) Rate of ATP production as calculated from glycolysis (n = 8), glucose oxidation (n = 8), and palmitate oxidation (n = 8) in the pre-and post-ischemic hearts perfused with 1.2 mM palmitate, 5 mM glucose, and 100 μU/ml insulin, (**b**) Percent contribution of each pathway to the total cardiac ATP production, (**c**) TCA cycle activity (acetyl-CoA produced for the TCA cycle) based on the calculation from glucose oxidation (n = 8), palmitate oxidation (n = 8), (**d**) Percent contribution of each pathway to TCA activity. A two-way ANOVA with Bonferroni correction for multiple comparisons was carried out for each panel in this figure. Data are expressed as mean ± SEM. ****P < 0.0001,***P < 0.001, **P < 0.01, *P < 0.05 compared to pre-ischemic baseline values.

ATP production rates and TCA cycle activity fully recovered in post-ischemic hearts (Fig. 3). However, the source of this ATP production changed during reperfusion. While fatty acid oxidation contributed to 90% of the total ATP production at baseline, this contribution was further elevated to 93% in reperfused ischemic hearts (Fig. 3a,b). Conversely, glycolysis and glucose oxidation initially accounted for only 4.9% and 6.1% of ATP production, respectively, before ischemia. However, following reperfusion, the contribution of glucose oxidation to ATP production was reduced to less than 2%, while the contribution from glycolysis slightly increased to 5.5% (Fig. 3a,b).

During reperfusion, palmitate oxidation provided 98.3 ± 0.9% of the acetyl-CoA for the TCA cycle, in contrast to 94.9 ± 1.6% in pre-ischemia. In contrast, glucose oxidation contributed only a minor proportion of the acetyl-CoA for the TCA cycle during reperfusion (1.63%, down from 5.1% in the pre-ischemic period). In summary, both overall ATP production and acetyl-CoA for the TCA cycle were significantly increased in the post-ischemic hearts primarily attributable to heightened fatty acid oxidation rate and (glycolysis for ATP).

### Ischemia and reperfusion do not alter NAD^+^ and NADH levels in the heart

**Figure 4:**
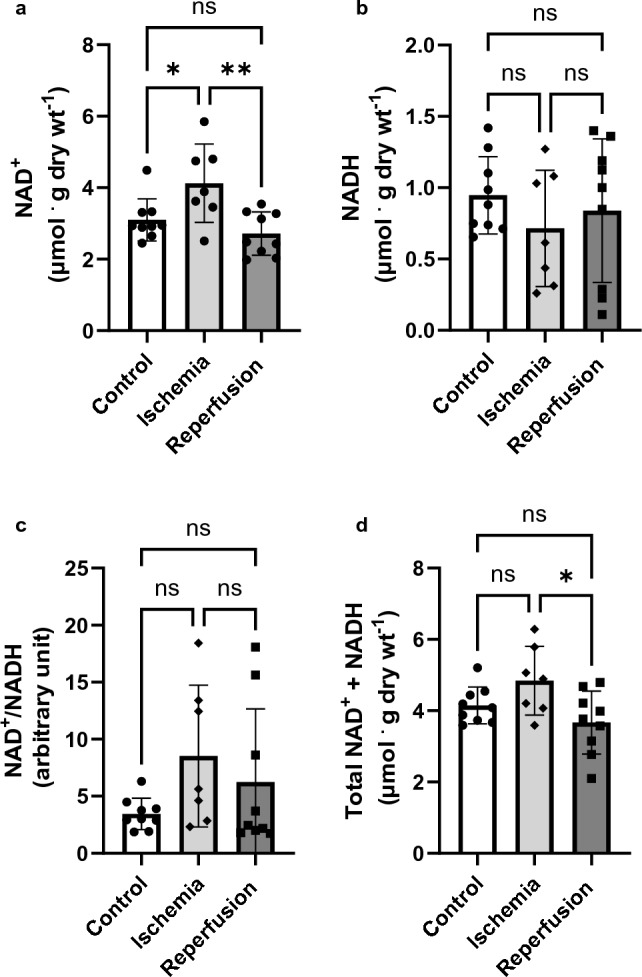
Unchanged NAD^+^ and NADH levels during and after ischemia. The NAD^+^ and NADH levels were determined from heart tissue snap-frozen at the end of ischemia and reperfusion using an Amplite™ Colorimetric NADH Assay Kit (n = 7–9 for each). (**a**) Cardiac NAD^+^ content before and after ischemia, (**b**) Cardiac NADH content before and after ischemia, (**c**) Ratio of cardiac NAD^+^ to NADH, and (**d**) Overall nucleotide content. A paired t-test was used for pre-and post-ischemic comparisons. Data are expressed as mean ± SD. **P < 0.01 *P < 0.05 compared to pre-ischemic values.

There was no significant difference in the NAD^+^ content between control (non-ischemic) hearts and reperfused hearts. However, NAD^+^ values were slightly lower in the post-ischemic hearts compared to controls, although this difference did not reach statistical significance (3.103 ± 0.6 vs 2.7 ± 0.6 μmol ^.^ g dry wt^–1^, p-value: 0.53). Similarly, the NADH levels also remained unaltered after ischemia (0.95 ± 0.3 vs 0.84 ± 0.50 μmol ^.^ g dry wt^–1^; p-value: 0.84). Likewise, the NAD^+^/NADH ratio was not substantially influenced by ischemia, although a slight increase was observed in post-ischemic hearts (3.5 ± 1.4 vs 6.2 ± 6.4). Furthermore, there was a reduction in the overall NAD^+^ plus NADH content in post-ischemic hearts compared to control hearts (Fig. 4a–d).

### Acetylation and succinylation status are not altered in post-ischemic heart

**Figure 5:**
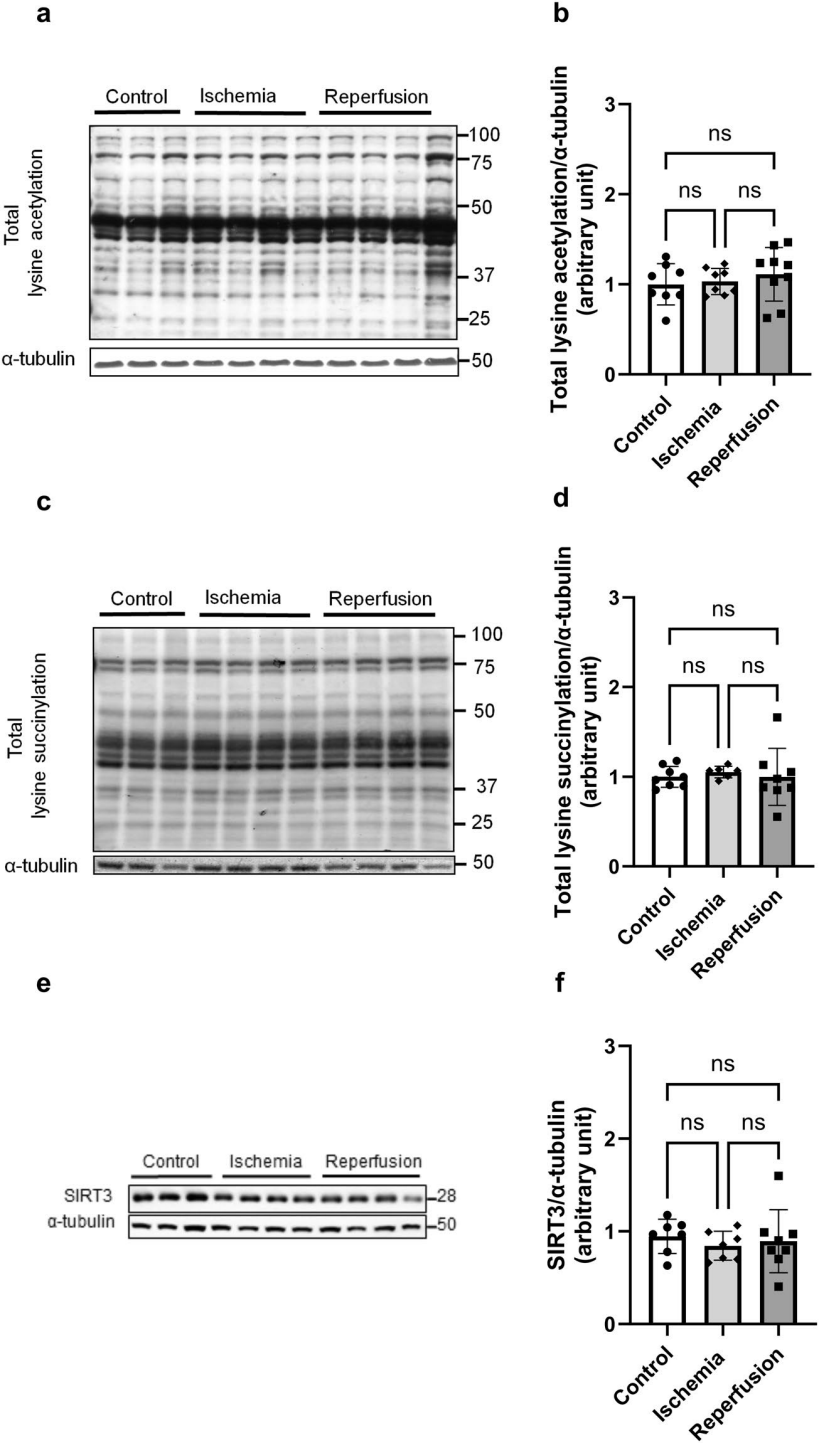
Global lysine acetylation and succinylation status are not affected by ischemia and reperfusion. Samples were collected from aerobic hearts (controls) at the end of the ischemic period (end of ischemia), or at the end of reperfusion. (**a**–**c**) Representative blots for total protein lysine acetylation, total protein lysine succinylation, and SIRT3, (**d**–**f**) Densitometry analysis for each blot (n = 7–9). A one-way ANOVA with multiple comparisons was done for each panel in this figure.

**Figure 6:**
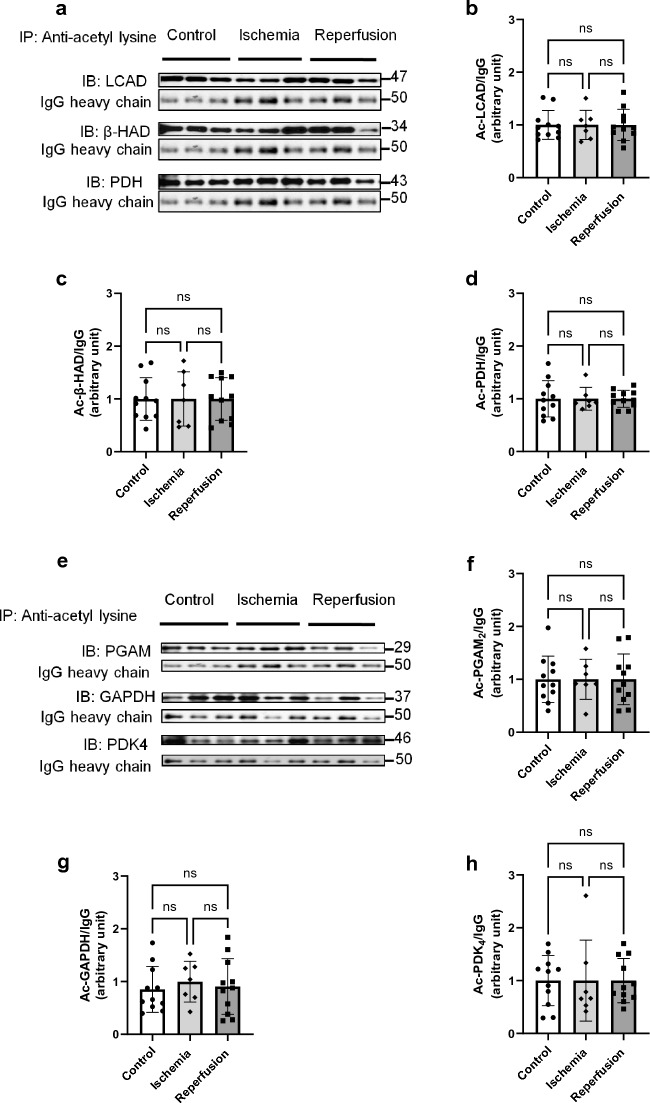
No change in the acetylation status of major glucose and fatty acid oxidation enzymes was observed in isolated working rat hearts after ischemia and reperfusion. The acetylation status of the individual cardiac metabolic enzymes was determined using the immunoprecipitation technique using an acetyl-lysine antibody. (**a**) Representative blots, and (**b**–**d**) Densitometry analysis of acetylated LCAD, ꞵ-HAD and PDH, (**e**) Representative blots, and (**f**–**h**) Densitometry analysis of acetylated PGAM2, PDK4 and GAPDH normalized to the IgG heavy chain as a loading control (n = 7–9). Data are presented as mean ± SD. Data were analyzed using one-way ANOVA with multiple comparisons. *p < 0.05 was considered statistically significant.

To determine whether the high fatty acid oxidation and decreased glucose oxidation in post-ischemic heart were associated with dysregulation of protein acetylation status, we analyzed the post-translational acetylation patterns of metabolic enzymes and proteins involved both in glucose and fatty acid metabolism. However, to our surprise, we found no significant differences in total acetylation (Fig. 5a,b) or succinylation status (Fig. 5c,d) between control, ischemic and post-ischemic hearts. We also assessed the expression of SIRT3 protein, the main mitochondrial deacetylase enzyme, in these groups. However, there was no alteration in SIRT3 levels among the groups (Fig. 5e,f). We further investigated whether the acetylation status is changed at the level of individual metabolic enzymes of fatty and glucose oxidation in response to ischemia and reperfusion. However, there were no significant differences in the acetylation status of two key enzymes of fatty acid oxidation, (long-chain acyl-CoA dehydrogenase (LCAD) and β-hydroxy acyl-CoA dehydrogenase (ꞵ-HAD), between the control and post-ischemic hearts. Similarly, we found no changes in pyruvate dehydrogenase (PDH) acetylation, the rate-limiting enzyme in glucose oxidation (Fig. 6a–h). We also examined the acetylation status of several glycolytic enzymes in non-ischemic and post-ischemic hearts. Only phosphoglycerate mutase 2 (PGAM2) and glyceraldehyde 3-phosphate dehydrogenase (GAPDH) were found to be acetylated, but again no significant changes in acetylation were observed in ischemic and post-ischemic hearts. We also evaluated the protein levels of each of these enzymes. However, no changes in protein levels were observed (Supplement Fig. S4). These results suggest that acetylation may not have a regulatory role in the metabolic changes observed in post-ischemic hearts.

## Discussion

Mitochondrial protein acetylation can have a significant impact on cardiac energy metabolism (see review^54^). In this study, we assessed for the first time the post-translational acetylation dynamics of cardiac metabolic enzymes and their functional relevance in post-ischemic hearts. Contrary to our expectations, we found no changes in either the global or individual metabolic enzyme acetylation status after ischemia or reperfusion. Nevertheless, ischemia did result in a significant increase in fatty acid oxidation and glycolysis rates during reperfusion, along with the inhibition of glucose oxidation. We further demonstrated that the contribution of glucose oxidation to total ATP production and acetyl-CoA supply to the TCA cycle was significantly decreased in hearts reperfused after ischemia. These metabolic changes were accompanied by an incomplete recovery of cardiac work and cardiac efficiency during reperfusion. In line with the unaltered acetylation status, both NAD^+^ and NADH contents were also not significantly changed in reperfused ischemic hearts compared to non-ischemic hearts.

Recently, the regulatory effects of lysine acetylation on the activities of cardiac metabolic enzymes have been elucidated in high-fat diet-fed and obese mice as well as in the hearts of diabetic animals^33,35,55^. In these studies, the hyperacetylation state is demonstrated to have dual effects: augmenting the activities of fatty acid oxidation enzymes including LCAD and ß-HAD, while concurrently repressing pyruvate dehydrogenase (PDH)^38^. Under these circumstances, it has also been speculated that acetyl-CoA generated from high rates of fatty acid oxidation drives the hyperacetylation of mitochondrial enzymes^56^. Likewise, increased concentrations of fatty acids in the bloodstream and enhanced utilization in the heart have been observed following myocardial I/R, both in experimental and clinical studies^14,16,17^. While the conventional explanation attributes the rise in catecholamine levels during ischemic stress to adipose tissue lipolysis and the mobilization of free fatty acids^57–59^, alternative findings suggest that the surge in plasma fatty acid levels may also be induced by heparin, particularly in the clinical setting^60,61^. Heparin acts on lipoprotein lipase (LPL) in the endothelium, releasing it into the circulation and breaking down lipoproteins to release free fatty acids. Consequently, some researchers argue that earlier studies might have overestimated plasma fatty acid levels if conducted without LPL inhibition^61,62^. Regardless, we used a high fatty acid concentration in the current study, similar to the levels seen in the hearts of HFD-fed and diabetic animals. However, despite the high rates of fatty acid oxidation in the post-ischemic heart in the current study, we did not observe any significant alteration in the acetylation status of LCAD, ꞵ-HAD and PDH enzymes.

Several potential factors could theoretically contribute to an altered acetylation status in response to myocardial ischemia and reperfusion. Firstly, high fatty acid ß-oxidation rates during reperfusion, as demonstrated in the current study, may lead to the accumulation of acetyl-CoA, which promotes the nonenzymatic acetylation of mitochondrial proteins, as described above. This notion was corroborated through a well-designed isotope tracing experiment conducted by Pougovkina and colleagues^63^. Using radioactively labeled palmitate, the authors demonstrated that the excess acetyl-CoA generated from high rates of fatty acid oxidation was sufficient to drive global protein hyperacetylation^63^. More recently, these findings were further reinforced through a more intricate heart failure model in mice by Deng et al. where they showed an increased incorporation of fatty acid-derived acetyl-CoA into acetylated proteins following a high-fat diet^64^. Notably, it is also evident that this increased acetylation of fatty acid oxidation enzymes under such circumstances augments their enzymatic activity. However, the causes underlying inconsistencies in the changes or regulatory roles of acetylation within metabolic enzymes across diverse heart failure pathologies (such as ischemic versus diabetic or high fat-induced heart failure) remain less clear.

Using cutting-edge high-resolution mass spectrometry techniques, acetyl-proteomics studies have unveiled a large number of hyperacetylated proteins enriched with myocardial metabolic enzymes and proteins in non-ischemic heart failure models^65,66^. IP with specific lysine acetyl antibody followed by western blotting is also an effective method to analyze differences in acetylation dynamics in response to various interventions. In this study, we used this approach to assess the acetylation changes of major regulatory enzymes and proteins in glycolysis, glucose oxidation and fatty acid metabolism, along with the determination of flux analysis for each pathway and ex vivo cardiac function measurements in post-ischemic hearts. This enabled us to directly determine the functional consequences of acetylation modifications on metabolic rates and functional recovery. Surprisingly, we found that myocardial lysine acetylation remain unaltered by myocardial ischemia or reperfusion, indicating that changes in the acetylation status of fatty acid oxidative enzymes may not contribute to the high fatty acid oxidation rates seen in the post-ischemic heart.

The high rates of cardiac fatty acid oxidation seen in obesity and diabetes are accompanied by an increase in the acetylation and activities of fatty acid oxidation enzymes^33,35,36^. Given this context, the lack of a concomitant increase in the acetylation of fatty acid oxidative enzymes amidst the high rates of fatty acid oxidation during reperfusion of ischemic hearts in the present study remains unclear. A plausible explanation for these disparities could be attributed to the presence of acidosis that occurs during ischemia. It is assumed that most mitochondrial protein acetylation occurs non-enzymatically in the presence of excessive acetyl-CoA and an alkaline pH^67^. However, a decline in intracellular pH during ischemia might impede the nonenzymatic acetylation process^11^.

Reduced mitochondrial oxidative phosphorylation rates during myocardial ischemia compromises the conversion of NADH to NAD^+^, a critical co-factor for sirtuins^68^. Accordingly, increased NADH levels accompanied by a decline in NAD^+^ may occur following ischemia^69,70^. This decrease in NAD^+^ levels, anticipated during both ischemia and reperfusion, could theoretically inhibit SIRT3 activity and thus could contribute to a hyperacetylation state. However, our observation contradicts this assumption as we observed no differences in either NAD^+^ or NADH levels during and after ischemia. Similar to our findings, other researchers have also reported unaltered NAD^+^ levels in hearts subjected to 20 min of ischemia ^71^. However, our results are in contrast to the previous studies, which reported a significant elevation in NADH level at the end of ischemia^72,73^ and in reperfusion^70^ and a decline in NAD^+^ values^74^. While further investigation is necessary to resolve these differences, various factors may contribute to these variable observations, including variation in the duration of ischemia, perfusion conditions, differences in timing and assay methods for NAD^+^/NADH measurements.

Some studies have revealed a reduced expression of SIRT3 protein in post-ischemic myocardial tissue which could potentially contribute to a hyperacetylation state^43,44,75^. However, we did not observe any changes in the protein level of SIRT3 at the end of ischemia or at the end of reperfusion. Regardless of this lack of changes in SIRT3 expression, SIRT3 activity could theoretically be decreased due to changes in NAD^+^ levels during ischemia and reperfusion. Although we did not directly assess SIRT3 activity, the unaltered levels of both NAD^+^ and NADH along with unchanged acetylation patterns suggest that SIRT3 activity may not be affected by ischemia and reperfusion. Previous studies have evaluated myocardial ischemia–reperfusion injury in mice deficient of SIRT3^45,46,76^. However, these studies produced mixed results. While some reported an increased susceptibility of SIRT3 KO mice to I/R injury^45,46^, others found no significant differences in the severity of myocardial injury in SIRT3 KO vs WT control mice^77^. Nevertheless, in all of these studies, neither the metabolic alterations nor the acetylation status of metabolic enzymes were directly assessed.

Similarly, previous studies have also shown that myocardial I/R may affect the activity and expression of SIRT1^78^. Several other studies also indicated that SIRT1 has a cardio-protective role during myocardial I/R by modulating oxidative stress, apoptosis and other pathways^79,80^. In addition, it is suggested that SIRT1 may regulate PGC-1α and PPARα activities, which in turn influences the transcription of genes related to fatty acid metabolism^81,82^. However, it is not clear whether SIRT1-mediated deacetylation affects acute energy metabolic changes during myocardial I/R. In our study, we didn’t analyze the SIRT1 levels and the acetylation status of SIRT1 targets in the nucleus, which may have an indirect effect on mitochondrial metabolic changes. However, considering the acute nature of our study, it is less likely that mechanisms mediated by SIRT1 may contribute the acute mitochondrial metabolic alterations seen in post-ischemic heart.

For decades, the altered myocardial energy substrate utilization has been recognized as a significant contributor to the impaired recovery of cardiac function and subsequent heart failure development in post-ischemic hearts. In line with early studies^11,29^, we observed an increase in fatty acid oxidation following ischemia accompanied by a decline in both cardiac work and cardiac efficiency. Significant efforts have been made to utilize cardiac energy substrate manipulation as an adjunct therapeutic avenue in post-ischemic heart failure. In agreement with this, several studies have demonstrated an improved cardiac efficiency and recovery in post-ischemic hearts through interventions such as inhibiting myocardial fatty acid oxidation using drugs such as etomoxir, perhexilne, trimetazidine, and malonyl CoA decarboxylase inhibitors, or by stimulating glucose oxidation with dichloroacetate (see Ref.^83^ for review). However, while many promising outcomes emerged from preclinical studies, the translation of these findings into routine clinical practices has been less successful, leaving room for the development of effective interventions to optimize cardiac energy metabolism in post-ischemic hearts. In our study, we investigated the roles of emerging mechanisms of post-translational protein acetylation modifications of cardiac metabolic enzymes as a new strategy to modulate the heart’s metabolic substrate utilization in post-ischemic heart. Although recent studies described the critical roles of dysregulated cardiac protein acetylation in myocardial metabolic disturbances and non-ischemic heart failure development (see Ref.^54^ for review), we found no changes in acetylation status despite perturbed cardiac energy metabolism in post-ischemic heart. The therapeutic potential of targeting protein acetylation in heart failure and other diseases is being researched. Several small molecule inhibitors and activators, acetyltransferases and deacetylases (sirtuins) have been developed and tested in preclinical studies to neutralize aberrant protein acetylation changes, thereby improving metabolic homeostasis and functional outcomes in heart failure and other diseases^84^. Despite this, our data suggest that drugs that inhibit hyperacetylation, such as SIRT3 activators, may not be a therapeutic approach to decreasing fatty acid oxidation in post-ischemic hearts. However, since increased lysine acetylation levels have been linked with the worsening of metabolic diseases such as diabetes and obesity as well as several cancer types, targeting protein lysine acetylation modification may be a potential therapeutic strategy in these disease states^85^.

### Limitations

There are some limitations in the current study. Firstly, we used an immunoprecipitation method to assess acetylation changes in key regulatory enzymes of glycolysis, glucose oxidation and fatty acid metabolism. Unlike acetyl-proteomics approaches, this method lacks the ability to identify specific acetylation sites. Additionally, the immunoprecipitation method is suboptimal for detecting the full spectrum of protein acetylation changes that could potentially occur in response to I/R. However, since there were no significant changes in global or major regulatory enzymes' acetylation status across our study groups, the relevance of identifying acetylation sites or other protein acetylation events becomes less important. Secondly, our study focused exclusively on cardiac fatty acid and glucose metabolism. However, it is worth noting that other fuel substrates, including branched-chain amino acids and ketone bodies, might also contribute to cardiac dysfunction and injury after myocardial I/R. Future studies on the role of these substrates in the recovery of cardiac function after I/R, as well as on acetylation dynamics, will be important. Thirdly, although our acute ex vivo myocardial I/R model is sufficient to study the short-term effects of I/R on protein acetylation, this may not fully represent the chronic and in vivo responses to I/R. Further studies on the chronic impact of in vivo I/R on enzymes or proteins regulating acetylation balance are necessary to fully understand the regulatory role of post-translation protein acetylation in myocardial I/R. Moreover, the studies described here were performed only in male rats. However, ischemia/reperfusion injury is a very relevant clinical condition in female patients. Therefore, additional studies are needed to examine the regulatory roles of acetylation on cardiac energy metabolism in post-ischemic hearts from female rats. Lastly, we used global no-flow ischemia to induce severe ventricular dysfunction, as indicated by the incomplete recovery of the mechanical function upon reperfusion. However, we didn’t assess directly the irreversible injuries after 30 min of ischemia other than troponin I level estimation at the end of the reperfusion. Due to the short frame of time, our protocol may not have significant effect on cardiac troponin I release. Additionally, it is also important to recognize that regional and low-flow ischemia are also relevant in vivo and clinical settings. The extent to which the severity of ischemic injury affects the results observed in our study needs further investigation.

## Conclusions

In our study, we revealed the following interesting observations. Firstly, we found increased fatty acid oxidation rates in post-ischemic hearts despite impaired recovery of contractile function and cardiac efficiency. Intriguingly, the acetylation status of enzymes involved in both fatty acid and glucose oxidation remain unchanged in post-ischemic hearts despite the high rates of fatty acid oxidation. This suggests that alterations in acetylation status might not exert a regulatory role in the altered rates of fatty acid oxidation in post-ischemic hearts. Furthermore, we observed that ischemia and reperfusion did not affect the NAD^+^ and NADH redox states. Overall, our study provides valuable insights into the role of lysine acetylation in regulating metabolic changes in post-ischemic heart.

### Supplementary Information

Supplementary Figures.

## Data Availability

The datasets used and/or analysed during the current study are available from the corresponding author upon reasonable request.
