# Supplementary material for: Protein lysine acetylation does not contribute to the high rates of fatty acid oxidation seen in the post-ischemic heart
Source: Sci Rep. 2024 Jan 12;14:1193. doi: 10.1038/s41598-024-51571-0 (PMC10786925; doi:10.1038/s41598-024-51571-0)

**Supplement Figure Legends:**

**Supplement Figure 1: Changes in fatty acid oxidation rates per unit cardiac work in pre-and post-ischemic hearts**

The graph was plotted to show the changes in fatty acid oxidation rate compared to cardiac work per unit time. Individual values on the y-axis were obtained by dividing fatty acid oxidation rates by cardiac work at each time point shown on the x-axis (n=5).

**Supplement Figure 2: Uncropped and full length images of western blots used in Figures 5**

a) Corresponds to Fig 5a, b) Corresponds to Fig 5c (α-tubulin for Fig 5c was original cut and saved in this form), c) Corresponds to Fig 5e.

**Supplement Figure 3: Uncropped and full length images of western blots used in Figure 6**

a) Corresponds to Fig 6a, (the same IgG loading control was used for Fig 6a), b) Corresponds to Fig 6e. LCAD: long-chain acyl-CoA dehydrogenase; ꞵ-HAD: β-hydroxy acyl-CoA dehydrogenase; PDH: pyruvate dehydrogenase; PGAM: phosphoglycerate mutase; GAPDH: glyceraldehyde 3-phosphate dehydrogenase; PDK4: pyruvate dehydrogenase kinase 4.

**Supplement Figure 4: Protein levels of acetylated cardiac metabolic enzymes detected by IP**

The protein levels of all metabolic enzymes was determined by western blot in the samples used for acetylation studies by IP (n=4). a) Immunoblot of metabolic enzymes subjected to acetylation study by IP, b-d) Quantification of densitometry from western blot, e) Immunoblot of metabolic enzymes subjected to acetylation study by IP, f-h) Quantification of densitometry from western blot. LCAD: long-chain acyl-CoA dehydrogenase; ꞵ-HAD: β-hydroxy acyl-CoA dehydrogenase; PDH: pyruvate dehydrogenase; PGAM: phosphoglycerate mutase; GAPDH: glyceraldehyde 3-phosphate dehydrogenase; PDK4: pyruvate dehydrogenase kinase 4.

**Supplement Figure 5: Uncropped and full length images of western blots used in supplement Figure 4**

a) Corresponds to supplement figure 4a, b) Corresponds to supplement figure 4b (β-HAD and PDH were probed on the same member after cutting it at about 37kDa position)

Supplement Figure 1


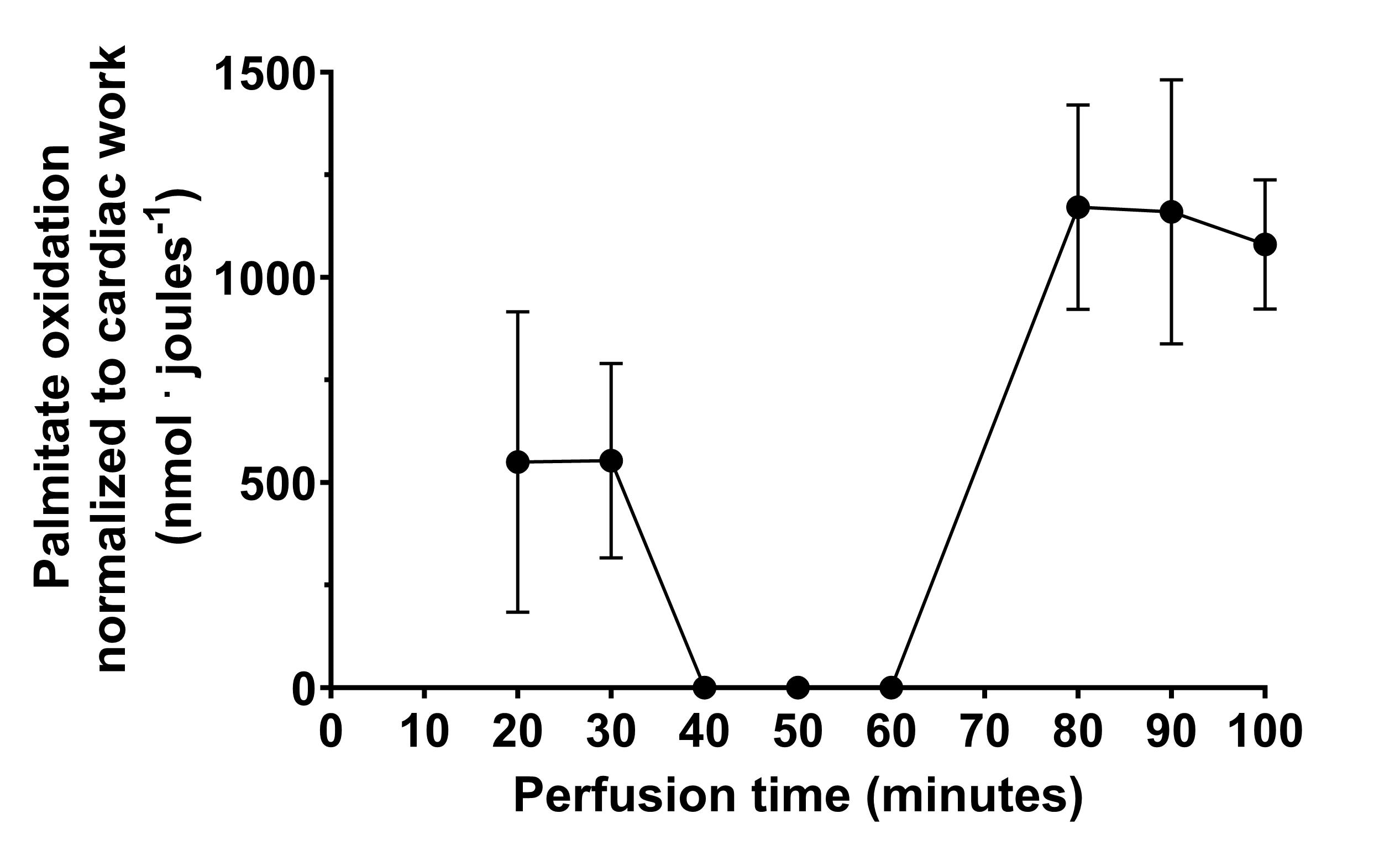


Supplement Figure 2


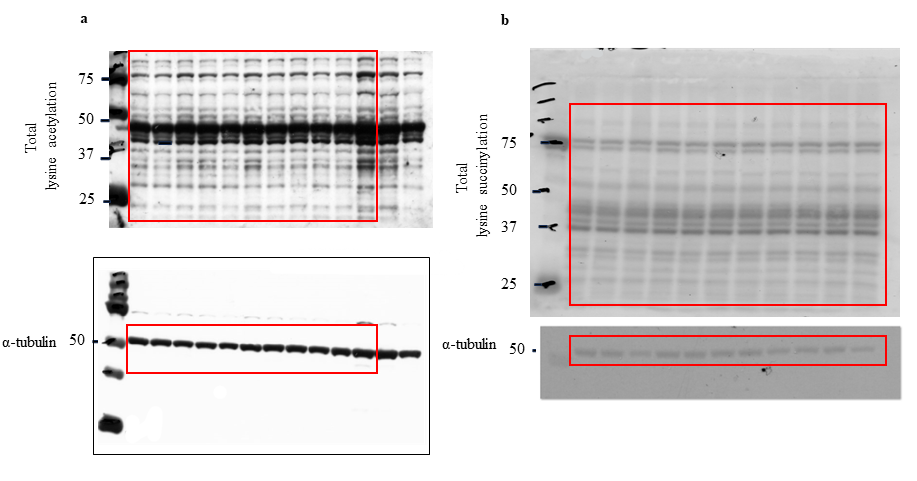


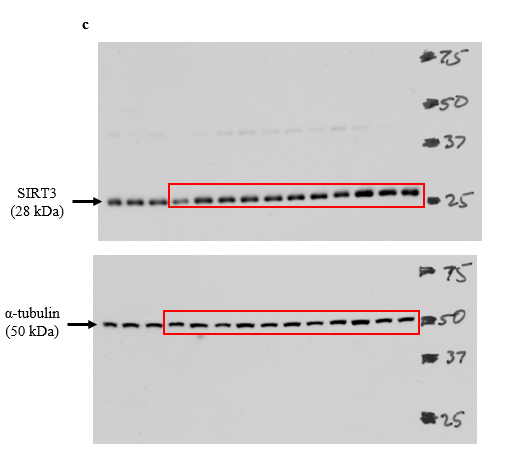


Supplement Figure 3


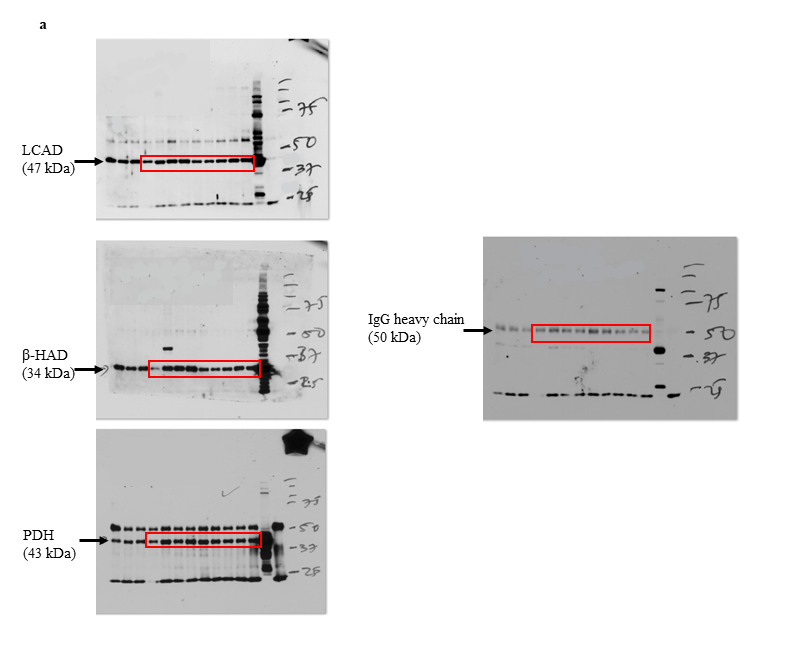


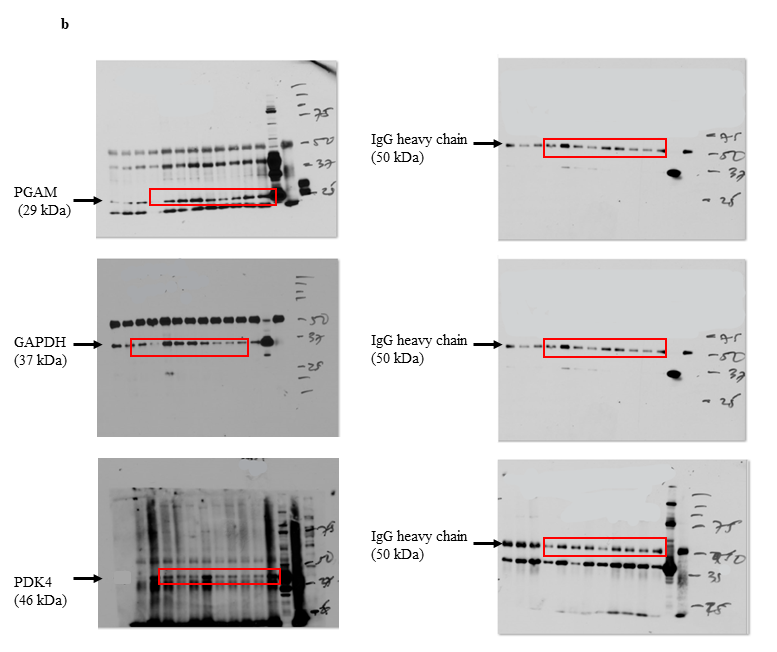


Supplement Figure 4

Supplement Figure 5


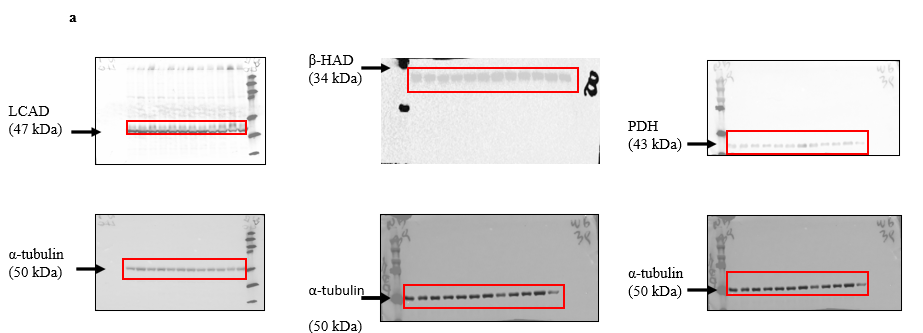


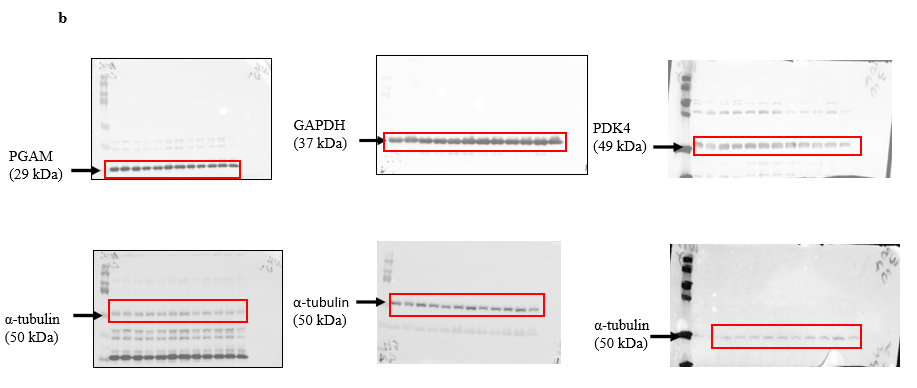

Supplement: Supplementary file 1 — Supplementary Figures. [file 41598_2024_51571_MOESM1_ESM.docx]
